# PMCNA_RS00975 activates NF-κB and ERK1/2 through TLR2 and contributes to the virulence of *Pasteurella multocida*

**DOI:** 10.3389/fcimb.2024.1469304

**Published:** 2024-10-15

**Authors:** Tenglin Xu, Mingxing Kou, Peili Cao, Benjin Liu, Yating Zheng, Qian Jiang, Jiasen Liu, Hongtao Kang, Mingfa Yang, Dongchun Guo, Liandong Qu

**Affiliations:** Division of Zoonosis of Natural Foci, State Key Laboratory of Veterinary Biotechnology, Harbin Veterinary Research Institute, Chinese Academy of Agricultural Sciences, Harbin, China

**Keywords:** *Pasteurella multocida*, PMCNA_RS00975, virulence factor, signaling pathway, proinflammatory cytokines

## Abstract

**Introduction:**

*Pasteurella multocida* is a pathogenic bacterium known to cause hemorrhagic septicemia and pneumonia in poultry. Reports have indicated that certain proteins, either directly involved in or regulating iron metabolism, are important virulence factors of *P. multocida*. Therefore, understanding virulent factors and analyzing the role of pro-inflammatory cytokines can help us elucidate the underlying pathogenesis.

**Methods:**

In this study, the PMCNA_RS00975 protein, a putative encapsuling protein encoded by a gene from a specific prophage island of the pathogenic strain *C48-1* of *P. multocida*, was investigated. To further explore the impact of the PMCNA_RS00975 protein on pathogenicity, a *PMCNA_RS00975* gene mutant of *P. multocida* strain *C48-1* was constructed using positive selection technology. Subcellular localization was performed to determine the location of the PMCNA_RS00975 protein within *P. multocida.* The recombinant protein PMCNA_RS00975 of *P. multocida* was soluble expressed, purified, and its role in pro-inflammatory cytokines was investigated.

**Results:**

The mutant exhibited significantly reduced pathogenicity in the mice model. Furthermore, subcellular localization indicated that the PMCNA_RS00975 protein was located at the outer membrane and expressed during infection of *P. multocida.* Additionally, our experiments revealed that recombinant PMCNA_RS00975 protein promotes the secretion of the IL-6 pro-inflammatory cytokines triggered by the TLR2 receptor via NF-κB and ERK1/2 signaling pathways in the macrophages.

**Discussion:**

This study identified a novel virulence factor in the *C48-1* strain, providing a basis for understanding the pathogenesis and directions for the development of attenuated vaccines against *P. multocida.*

## 1 Introduction

*Pasteurella multocida* (*P. multocida*) is an encapsulated, gram-negative coccobacillus that belongs to the genus *Pasteurella* and causes several animal and human infections, including avian cholera, swine pneumonia, bovine hemorrhagic sepsis, and rabbit hemorrhagic sepsis (Lu et al., 2023). *P. multocida* infections in livestock industries have been reported to have high mortality rates and production losses, resulting in considerable economic loss and hardship, especially in resource-poor regions (Mostaan et al., 2020). The serotypes of *P. multocida* are largely distinguished by capsular antigens as A, B, D, E, and F, whereas lipopolysaccharide (LPS) antigens are divided into 16 types (Harper et al., 2011). Researchers have developed mouse models to explore the mechanism of pathogenesis of *P. multocida*. In addition, vaccination plays an important role in improving the health and welfare of livestock and preventing animal transmission. However, currently, available attenuated and inactivated vaccines cannot sufficiently provide cross-protection against all serotypes (Guan et al., 2021; Abbasi et al., 2023; Casalino et al., 2024). Moreover, the interaction mechanism between bacteria and host remains unclear.

*P. multocida* uses iron regulation and acquisition as the key biological process for their survival and pathogenesis in the host (Jatuponwiphat et al., 2019). It has been reported that 2.5% of the total genes (53 genes) in the Pm70 genome and 2.1% of the total genes (46 genes) in the HB01 genome are predicted to encode proteins involved in iron metabolism (May et al., 2001; Peng et al., 2016; Jatuponwiphat et al., 2019). Several operons and genes associated with iron metabolism have been proposed, such as TonB-dependent transporter (TBDT) (Silale and van den Berg, 2023), *afuCBA*, f*ecABCDE*, *yefABCD*, and *fbpABC* operons involved in transport, the *fur* and *hgbA* gene and associated with ferric utilization (Peng et al., 2016). The ferritin family includes three subfamilies, namely, canonical ferritin, heme-containing bacterioferritin, and DNA-binding proteins (Andrews, 2010). Recently, the encapsulated ferritin (EncFTN) has been identified as a new member of the ferritin family that is involved in iron storage and is widely distributed in viruses, bacteria, archaea, and eukarya (Giessen, 2022).

Toll-like receptors (TLRs), functioning as pattern recognition receptors, play critical roles in early innate recognition and the expression and release of different cytokines by host against invading microbes (Wang et al., 2020). Several molecules in bacteria such as peptidoglycans, lipoarabinomannan, zymosan, and lipoproteins are recognized by TLR2 of immune cells, causing the activation of downstream nuclear factor kappa-B (NF-κB) and mitogen-activated protein kinase (MAPK) signaling pathways to consequently induce inflammatory cytokines (Chandler and Ernst, 2017; Lundstedt et al., 2021; Mitchison-Field and Belin, 2023). LPS derived from gram-negative bacterial cell walls function as major pathogen-associated molecular patterns (PAMPs) and a natural TLR4 ligand, leading to the activation of downstream NF-κB (Otten et al., 2021; Wang et al., 2023). The chip technology and transcriptomic technology have been used to detect TLRs, NF-κB, MAPK, tumor necrosis factor (TNF), janus kinase/signal transducers, and activators of transcription (JAK-STAT) and NOD1/2-like receptors in the spleen, liver, and lungs of mice infected with *P. multocida*. These molecules cause the release of IL-1β, IL-6, TNF-α, and other cytokines (Priya et al., 2017; Wu et al., 2017). In addition, PM0442 is a lipoprotein located at the outer membrane of *P. multocida*, encoding the Pm0442 protein that activates the NF-κB and MAPK signaling pathways through TLR2 to secrete IL-1β, TNF-α, and IL-6 (He et al., 2020).

Whole-genome sequencing, signature-tagged mutagenesis, and comparative genomics have been employed to study different virulence factors of *P. multocida* in the pathogenic mechanism (Guo et al., 2012; Chitarra et al., 2018; Peng et al., 2019). We have previously demonstrated using whole-genome sequencing and bioinformatics analysis in *P. multocida* that the *C48-1* strain contains one complete unique region of 44.8 kb—predicted to be a putative prophage with similarity to a *Mannheimia* lambda-like bacteriophage (Cao et al., 2017). The prophage island of the *C48-1* strain contains 55 CDSs (44.8 kb, *PMCNA_RS00890*-*PMCNA_RS01205*), mostly encoding phage-related proteins, and other unknown functional proteins (Cao et al., 2017; Peng et al., 2019). The non–phage-related proteins PMCNA_RS01145 can induce the release of pro-inflammatory cytokines TNF-α and IL-1β by mouse peritoneal macrophages (Xu et al., 2023). However, the PMCNA_RS00975 is a non–phage-related protein in the lysogenic phage gene cluster of the pathogenic strain *C48-1* of *P. multocida*. Bioinformatics analysis has speculated PMCNA_RS00975 as an encapsulating protein; however, its virulence in *P. multocida* and induced pro-inflammatory cytokines by macrophages are not yet known. In this study, we evaluated the *PMCNA_RS00975* gene delete mutant of *P. multocida C48-1* strain. The PMCNA_RS00975 protein was located at the outer membrane of *P. multocida*, triggering the secretion of IL-6 pro-inflammatory cytokines by macrophages through the TLR2-NF-κB and MAPK signaling pathways. We believe this study will contribute to understanding the pathogenicity and infection mechanism of *P. multocida*.

## 2 Materials and methods

### 2.1 Strains, plasmids, and cell culture

The bacteria strains and plasmids used in this study are listed in **Table 1**. The pathogenic strain *C48-1* (A:1) of *P. multocida* was isolated from chicken with fowl cholera in Jiangsu, China, in 1953 and was stored in our laboratory. *P. multocida C48-1* strain (A:1) was cultured in tryptic soy broth (TSB; Difco Laboratories, USA) or tryptic soy broth agar (TSA) medium containing 5% fetal bovine serum (FBS; Sigma-Aldrich, USA) at 37°C. *Escherichia coli* DH5α and BL21 (DE3) were cultured in the lysogeny broth (LB; Difco Laboratories, USA) medium at 37°C. Mouse macrophages RAW264.7 were cultured in the Dulbecco’s modified Eagle medium (DMEM; Sigma-Aldrich, USA) containing 10% FBS and under 5% CO_2_. Wild-type C57BL/6N, TLR2,^−/−^ and TLR4^−/−^ mice were injected intraperitoneally with 4% thioglycolate broth (Sigma-Aldrich, USA). After 3 days of induction, the peritoneal macrophages were collected and cultured in the Roswell Park Memorial Institute (RPMI; Sigma-Aldrich, USA) 1640 medium with 10% FBS under 5% CO_2_ for 2 h. The C57BL/6N mice were purchased from the Charles River Laboratories (Beijing, China). TLR2^−/−^ and TLR4^−/−^ with a C57BL/6N background were purchased from Cyagen Biosciences (Suzhou, China). Mice were maintained under specific pathogen-free (SPF) conditions in a standard animal care facility (Laboratory Animal Center, Harbin Veterinary Research Institute, Harbin, China). All animal experiments were performed according to the animal protocols approved by the Laboratory Animal Ethical Commission of Harbin Veterinary Research Institute, CAAS (HVRI-IACUC-2019-214).

**Table 1:** Summary of bacterial strains and plasmid used in this study.

Group	Names	Characteristics and functions	Sources/references
Bacterial strains	*P. multocida C48-1*	Serotype A:1, Chicken; Jiangsu, China	China Veterinary Culture Collection Center
	*C48-ΔPMCNA_RS00975*	*PMCNA_RS00975* deletion mutant	This study
	*C48-ΔPMCNA_RS00975C*	Complemented strain of *C48-ΔPMCNA_RS00975C*	This study
	*Escherichia coli* DH5α	Cloning host for recombinant vector	Lab collection
	*E. coli* BL21	Expression host for recombinant protein	Lab collection
Plasmids	pPRO-EX-HTb	Expression vector; *Amp* ^R^	Invitrogen
	pPRO-EXHTb- *PMCNA_RS00975*	Expression vector for the rPMCNA_RS00975 protein	This study
	pBC-SK	*Cm* ^R^ PUC^ori^	Stratagene
	pUC-4K	Km^R^	Amersham
	pBC- *PMCNA_RS00975*M	Derived from pBC-SK used to knock out *PMCNA_RS00975* gene in *C48-1*; *Kan* ^R^	This study
	pAL99*spec* ^R^	Shuttle vector between *E. coli* and *P. multocida*; *Spec* ^R^	This study
	pAL99*spec* ^R^- *PMCNA_RS00975*	Insertion of the *PMCNA_RS00975* gene expression cassettes into pAL99*spec* ^R^.	This study

Amp^R^, Ampicillin-resistant; Cm^R^, Chloramphenicol-resistant; Kan^R^, kanamycin-resistant; spec^R^, spectinomycin-resistant.

### 2.2 Antibodies and reagents

The following antibodies were used: antibodies against ERK1/2, p38, jun N-terminal kinase (JNK), p65, p-ERK1/2, p-p38, p-JNK, and p-p65 were purchased from Cell Signaling Technology (USA); β-actin was purchased from Yeasen (Shanghai, China). Antibodies against TLR2 and TLR4 were purchased from Novus Biological (USA). Mouse IL-6 enzyme-linked immunosorbent assay (ELISA) kits used were purchased from Neobioscience (China). The p38 inhibitor (SB203580), ERK1/2 inhibitor (PD98059), JNK inhibitor (SP600125), and NF-κB inhibitor (BAY 11-7082) were purchased from Sigma-Aldrich (USA).

### 2.3 Homology analysis of the PMCNA_RS00975 protein

The PMCNA_RS00975 amino acid sequences of *P. multocida* strain *C48-1* (GenBank Accession numbers: GCA_004286945.1) were aligned with the sequences of 17 homologous proteins from other bacteria (**Supplementary Table S1**) using the Basic Local Alignment Search Tool (BLAST). These protein sequences were aligned using the ClustalW program using the Molecular Evolution Genetics Analysis (MEGA) 11 software (10.1093/molbev/msab120). The phylogenetic tree of these proteins was generated using a neighbor-joining method.

### 2.4 Construction of *P. multocida PMCNA_RS00975* deletion mutant

The upstream and downstream sequences of the *PMCNA_RS00975* gene (999 bp) (GenBank accession number: OAZ06802.1) of the *C48-1* strain were amplified by PCR using primers PPm00975-up-F/R and PPm00975-down-F/R (**Table 2**). Based on the plasmid pUC-4K, the Kanamycin (*Kan*) resistance gene expression cassettes were amplified using the Kan-F/R primers (**Table 2**) and digested with *EcoR I* and *BamH I*. Afterward, the Kan resistance gene expression cassette was ligated to the vector pBluescript-SK (+) with the same digestion treatment to construct the recombinant plasmid pBC-*PMCNA_RS00975*M.

**Table 2:** Primer sequences used in this study.

Primer name	Sequence (5’→3’)	Enzyme sites/purpose
PPm00975-up-F	ACGCGTCGACTCTTATCAGCACTCAATGCC	*Sal* I
PPm00975-up-R	CGGAATTCTTACCCTCAATTAATCCACC	*Eco*R I
PPm00975-down-F	CGGGATCCCGAAACAGACGATTACAACT	*Bam*H I
PPm00975-down-R	TTCGAGCTCGCTACAGCTTCCCTACCTAC	*Sac* I
PPm00975-EF	CCCGGATCCTTAGCGAAAGCTGTTGCAGG	*Bam*H I
PPm00975-ER	CGGCTCGAGTTCGCTAGTTTCGCCAAG	XhoI
KMT1T7	CCGCTATTTACCCAGTGG	For identification of the *P. multocida*
KMT1SP6	TGTAAACGAACTCGCCAC	
Kan-F	TGGAATTCGGGGGGGGGGGAAAGCCAC	*Eco*R I
Kan-R	CGGGATCCGGGGGGGGGGGCGCTGAGGTCTG	*Bam*H I
Spec-F	TTGCATATGATGTCATCAGCGGTGGAG	*Nde I*
Spec-R	CCCCATATGTCATGAGATTATCAAAAAG	*Nde I*
PPm00975-JD-F	CTAATGGAGAAAACATAATGA	For identification of the mutant
PPm00975-JD-R	TATAGTCGCCTTTTTTAGCCT	
TLR2-F	GCTGGCGACCGGGAAGTTCG	For qRT-PCR assay
TLR2-R	TCTCCTGCCAGTGACCGCCT	
TLR4-F	ATGGCATGGCTTACACCACC	For qRT-PCR assay
TLR4-R	GAGGCCAATTTTGTCTCCACA	
GAPDH-F	AGGTCGGTGTGAACGGATTTG	For qRT-PCR assay
GAPDH-R	TGTAGACCATGTAGTTGAGGTCA	

Restriction enzyme cutting sites are marked by underlined letters.

The isogenic *PMCNA_RS00975* gene mutant was constructed using the method previously described (Aranda et al., 2010). Briefly, the plasmid pBC-*PMCNA_RS00975*M was electrotransformed into cells of the strain *C48-1*, and spread onto TSA containing 100 μg/mL Kan and incubated for 72 h at 37°C to select recombination single-crossover. The selected Kan mutant was spread on the chloromycetin (*Cm*)- and Kan-resistant media, and the Cm-sensitive but Kan-resistant mutant was selected to be the homologous recombination double-crossover. The primers PPm00975-JD-F/R and Kan-F/R (**Table 2**) were used to identify the double-crossover mutant. The successfully identified double-crossover mutant was termed *C48-ΔPMCNA_RS00975* and identified using PCR with primers KMT1T7/KMT1SP6. The identified correctly *C48-ΔPMCNA_RS00975* mutant strain was subcultured for 20 generations, and the *Kan* resistance gene was detected every five generations to identify its genetic stability.

For producing a complemented strain of the mutant, spectinomycin (*Spec*) resistance gene expression cassettes were amplified using the *Spec*-F/R primers (**Table 2**) and inserted into the endonuclease *Nde I* of plasmid pAL99 to generate the plasmid pAL99*spec* ^R^ (Harper et al., 2003; Jiang et al., 2015). Simultaneously, the *PMCNA_RS00975* gene expression cassettes comprising a 134-bp upstream segment, a 178-bp downstream segment, and 999-bp the *PMCNA_RS00975* were amplified from the genomic DNA of the wild-type strain using PCR using the primers PPm00975-EF/R (**Table 2**) and digested with *BamH I* and *Xho I*. Afterward, they were cloned into the shuttle plasmid pAL99*spec* ^R^ for constructing the recombinant plasmid pAL99*spec* ^R^-*PMCNA_RS00975*. Further, the recombined plasmid pAL99*spec* ^R^-*PMCNA_RS00975* was electrotransformed into the *C48-ΔPMCNA_RS00975* mutant under the same conditions as described above. Next, the pAL99*spec* ^R^-*PMCNA_RS00975* plasmids were transformed into the *C48-ΔPMCNA_RS00975* mutant strain to generate the complemented strain *C48-ΔPMCNA_RS00975C*. Next, the *C48-1* wild-type, *C48-ΔPMCNA_RS00975* mutant, and complemented strain *C48-ΔPMCNA_RS00975C* were cultured in cultured in TSB medium containing 5% fetal bovine serum with or without 100μM 2,2’-dipyridyl (iron-restrictive medium) at 37°C for 8 h, following which the OD_600nm_ values were measured. The sequences of the primers synthesized by Sangon (Shanghai, China) are shown in **Table 2**.

### 2.5 Virulence assessment in mice

To assess the function of *PMCNA_RS00975* in the virulence of *P. multocida*, C57BL/6N mice (6–8-week-old) were infected with the wild-type, mutant, or complemented strains via intraperitoneal injection at a dose of 5 × 10^1^ to 5 × 10^5^ CFUs (*n* = 10 per group). The virulence of the wild-type, mutant, and complemented strains was compared using the survival curve of mice and mortality on the 7th day post-infection. Mice showing severe clinical signs were considered moribund and were humanely killed.

Next, the function of *PMCNA_RS00975* in the virulence of *P. multocida* was confirmed by incubating the wild-type *C48-1*, *C48-ΔPMCNA_RS00975* mutant, and complemented strain *C48-ΔPMCNA_RS00975C* with RAW264.7 cells for 3 h, 6 h, 9 h, and 12 h at a dose of 10 CFUs. The expression of IL-6 from cell supernatant was collected and detected using a mouse IL-6 ELISA kit.

### 2.6 Purification of the recombinant PMCNA_RS00975 protein

The *PMCNA_RS00975* gene was amplified using PPm00975-EF/R and cloned into plasmid pPRO-EX-HTb to construct a recombinant plasmid pPRO-EXHTb-*PMCNA_RS00975*. The recombinant plasmid pPRO-EXHTb-*PMCNA_RS00975* was transformed into *Escherichia coli* BL21 (DE3) competent cells with 0.5 mM isopropyl β-D-1-thiogalactopyranoside (IPTG; Tiangen, China) at 16°C. The soluble protein was purified according to the manufacturer’s protocol of the Ni-NTA His-Bind Resin Affinity Kit and analyzed by sodium dodecyl sulfate-polyacrylamide gel electrophoresis (SDS-PAGE). Next, the purified recombinant PMCNA_RS00975 (rPMCNA_RS00975) proteins were dialyzed in PBS and analyzed using the anti-His monoclonal antibody (Thermo, USA) or the anti-rPMCNA_RS00975 mouse serum. The anti-rPMCNA_RS00975 mouse serum was prepared according to the previous method (Xu et al., 2023).

To determine if the expression of PMCNA_RS00975 protein by *P. multocida C48-1* strain elicited the production of antibodies during infection, we performed western blotting. The rabbit antiserum of *P. multocida C48-1*, produced in our laboratory, was used as the primary antibody to analyze the immunogenicity of the PMCNA_RS00975 protein produced by infection with *P. multocida C48-1*.

For western blotting analysis, the rPMCNA_RS00975 protein was transferred onto polyvinylidene fluoride membranes (Merck, Millipore, USA) and blocked with 5% skim milk overnight at 4°C. Anti-rPMCNA_RS00975 serum from mice or anti-*P. multocida C48-1* serum from rabbits was used as the primary antibody, and the IRdye800CW-conjugated goat anti-mouse IgG antibody or IRdye800CW-conjugated goat anti-rabbit IgG antibody was used as the secondary antibody. Subsequently, membranes were scanned and visualized by the LI-COR Odyssey CLx imaging system (LI-COR Biotechnology, USA).

### 2.7 Subcellular localization of the PMCNA_RS00975 protein

The subcellular location of PMCNA_RS00975 protein was located using a previously described method (Bose and Taylor, 2005). Briefly, the *C48-1* strain was cultured in the TSB medium containing 5% bovine serum at 37°C for 8 to 10 h and treated with polymyxin B sulfate (10 mg/mL) in PBS for 10 min on ice to release periplasmic contents. The residual cells in culture supernatants were removed through a 0.45 μm-pore size membrane. Spheroplasts were separated from the soluble periplasmic fraction by centrifugation at 10,000× g for 10 min. The spheroplast fraction was resuspended in 1× SDS-PAGE loading buffer, and the supernatant containing the periplasmic contents was diluted with 2× SDS-PAGE loading buffer. To obtain total membranes, the spheroplast fraction was resuspended in 10 mM Tris-Cl (pH 8.0) and sonicated to break open the cells. After low-speed centrifugation at 10,000× g at room temperature (RT) for 10 min to remove any unbroken cells, the lysate was centrifuged at 100,000×g at 4°C for 30 min to pellet the membrane fraction. The inner and outer membranes were differentially solubilized as follows. The membrane pellet was resuspended in 10 mM Tris-Cl–100 mM NaCl containing 2.5% Sarkosyl, held at RT for 30 min, and centrifuged at 200,000× g at RT for 1 h. The supernatant was retained as the inner membrane fraction, and the pellet containing the outer membrane was resuspended in 200 μL of 10 mM Tris-Cl (pH 8.0). Proteins were estimated using the Bicinchoninic Acid (BCA) Protein Assay Kit (Tiangen Biotechnology, China). Equal protein amounts (10 μg) from each fraction were subjected to western blotting using the anti-rPMCNA_RS00975 mouse serum, and IRdye800CW-conjugated goat anti-mouse IgG antibody (LI-COR Biotechnology, USA) was used as the secondary antibody. Subsequently, the membranes were scanned and visualized by the LI-COR Odyssey CLx imaging system (LI-COR Biotechnology, USA).

### 2.8 Peritoneal macrophage isolation and culture

The peritoneal macrophages of TLR2^−/−^ and TLR4^−/−^ with a C57BL/6N mice background were isolated using the method described previously (Xu et al., 2023). The peritoneal macrophages were seeded into 24-well microplates at a density of 3 × 10^5^ cells/well and incubated at 37°C with 5% CO_2_.

### 2.9 Cytokine ELISAs

RAW264.7 cells or isolated peritoneal macrophages were seeded into 24-well plates at a density of 2 × 10^5^ cells/well and stimulated with the rPMCNA_RS00975 protein or LPS (Sigma-Aldrich, USA) for 6 h. To confirm whether the purified protein was contaminated by LPS, the rPMCNA_RS00975 protein was boiled to ensure no residual LPS remains in the sample. In experiments designed to block the MAPK and NF-κB signaling, RAW264.7 cells were pretreated for 30 min at 37°C with inhibitors of p38 (20 μM), ERK1/2 (20 μM), JNK (20 μM), or NF-κB (20 μM) before the rPMCNA_RS00975 protein exposure as described above. In experiments designed to block the TLR signaling, peritoneal macrophages were pretreated for 1 h at 37°C with an antibody against TLR2 (5 μg/mL) or TLR4 (5 μg/mL). Simultaneously, peritoneal macrophages isolated from TLR2^-/-^, TLR4^−/−^, and wild-type mice were incubated with the rPMCNA_RS00975 protein. In the experiments designed to verify the neutralizing activity of the anti-rPMCNA_RS00975 mouse serum, the rPMCNA_RS00975 protein was pre-incubated with anti-rPMCNA_RS00975 mouse serum at 37°C for 1 h, then the mixture was exposed to peritoneal macrophages isolated from TLR2^-/-^, TLR4^−/−^, and wild-type mice for 6 h. Next, the cell culture supernatants were collected, and the expression of IL-6 was measured using the ELISA Kit.

### 2.10 Quantitative real-time PCR

The extraction/quantification of RNA from RAW264.7 cells was performed according to the previous method (Xu et al., 2023). RAW264.7 macrophages were incubated with the rPMCNA_RS00975 protein for 3 h. In the experiments designed to identify the TLRs involved in the induction of IL-6 cytokine by the rPMCNA_RS00975 protein, the rPMCNA_RS00975 protein was pre-incubated with mouse anti-serum at 37°C for 1 h, then the mixture was exposed to RAW264.7 cells for 3 h. Next, RNA levels of the analyzed genes were normalized to the amount of GAPDH present in each sample. The sequences of the qRT-PCR primers are listed in the **Table 2**.

### 2.11 Western blot analysis

Western blot analysis was performed using the previous method established in our laboratory (Xu et al., 2023). Briefly, RAW264.7 cells were seeded into 12-well plates at a density of 5 × 10^5^ cells/well. The cells were incubated at 37°C with 5% CO_2_ and stimulated with the purified rPMCNA_RS00975 for various time intervals or various doses. Next, the stimulated cells were lysed and centrifuged to collect the supernatant. Equal protein concentrations were separated by SDS-PAGE and severally detected with the primary antibodies against p-ERK1/2, p-p65, and β-actin. Subsequently, the IRdye800CW-conjugated anti-rabbit IgG (LI-COR Biotechnology, USA) or anti-mouse IgG antibodies were used as the secondary antibodies to visualize by the LI-COR Odyssey CLx imaging system. The signal intensity values in western blotting were quantified with the ImageJ software (National Institutes of Health, USA).

### 2.12 Statistical analysis

Statistical analysis was carried out using GraphPad version Prism 6.0 (GraphPad Software, USA). All data from experiments repeated was represented at least thrice are presented as the mean ± standard (SD) deviation. Differences in the groups were analyzed by corresponding *t*-test based on the Gaussian distribution and variance analysis. The *p-*value < 0.05 was considered significant.

## 3 Results

### 3.1 PMCNA_RS00975 is predicted as an encapsulin protein

**Figure 1:**
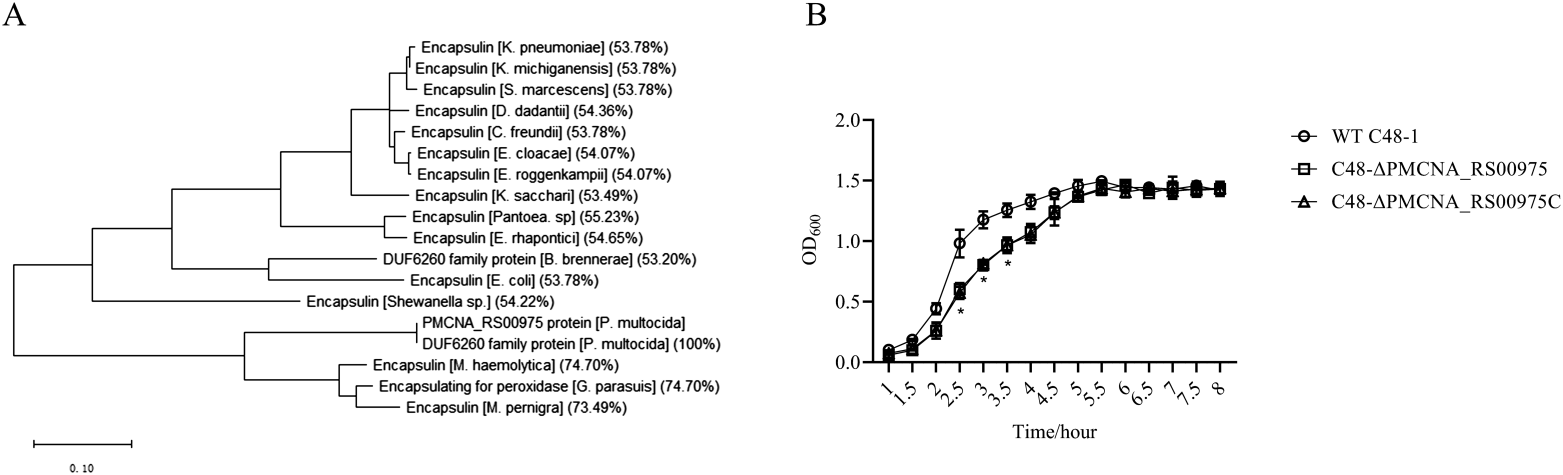
Phylogenetic tree of the *P. multocida* PMCNA_RS00975 protein with 17 homologs from others bacteria. **(A)** Numbers in parentheses denote identity with the PMCNA_RS00975 protein based on amino acid sequences. **(B)** The growth curves of *C48-1*, the *C48-ΔPMCNA_RS00975* mutant, and the complemented strain *C48-ΔPMCNA_RS00975C* in iron-restrictive medium (TSB+100μM 2,2’-dipyridyl) at 37°C for 8 h.

Amino acid sequences of these homologous genes exhibited more than 50% identity with that of the PMCNA_RS00975 from *P. multocida*. These homologous genes are mostly annotated as a putative encapsulin protein, also annotated as the DUF6260 family protein and encapsulating for peroxidase. The phylogenetic tree based on the deduced amino acid sequences of homologous genes revealed that the PMCNA_RS00975 protein belonged to the big branch neighbors, a putative encapsulating protein amino acid from *M. pernigra* and *G. parasuis*, and identity was 73.49% and 74.70%, respectively (**Figure 1A**). These results suggested that the PMCNA_RS00975 protein expressed from these homologous genes belonging to the clade and played a role in the same manner as *P. multocida* encapsulin and might be involved in iron metabolism. Additionally, the *C48-ΔPMCNA_RS00975* mutant showed growth retardation (2.5~3.5 h) compared to the wild-type strain in iron-restricted medium, which further indicates that the PMCNA_RS00975 protein is involved in the iron metabolism of *P. multocida* (**Figure 1B**).

### 3.2 Identification of the *PMCNA_RS00975* gene deletion mutant strain

To evaluate the function of *PMCNA_RS00975* in the virulence of *P. multocida*, the isogenic *PMCNA_RS00975* mutant *C48-ΔPMCNA_RS00975* was constructed based on positive selection techniques (**Supplementary Figure S1A**). Further, the *PMCNA_RS00975* gene of the 20th generation mutant was identified using PCR and DNA sequencing (data not shown). The recombinant plasmid pAL99*spec* ^R^-*PMCNA_RS00975* was used to electrotransform the mutant to obtain the complemented strain, which was identified using PCR (**Supplementary Figure S1B**). Further, the *C48-ΔPMCNA_RS00975* mutant exhibited no growth difference rate compared with the wild-type strain at cells/1 h to cells/8 h (**Supplementary Figure S1C**).

### 3.3 *PMCNA_RS00975* contributes to the virulence of *P. multocida* in mice model

**Figure 2:**
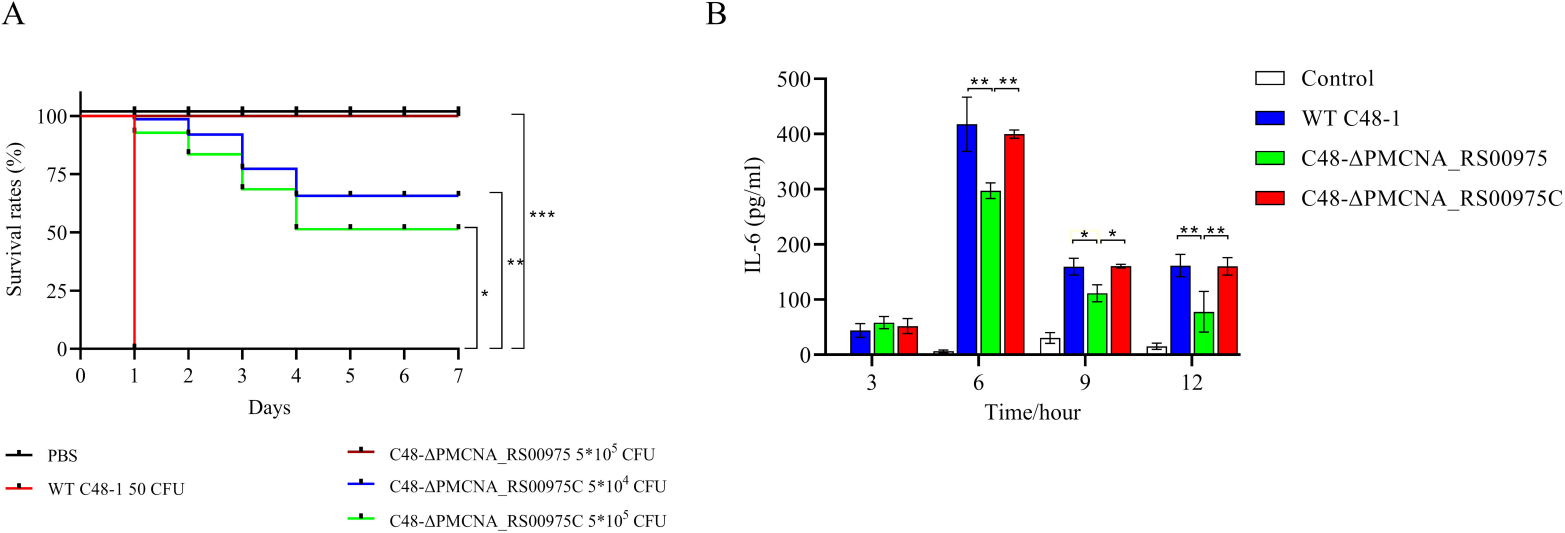
Virulence assessment of *P. multocida* in C57BL/6N mice model. **(A)** Survival curves of each group of mice (*n* = 10) exposed to the wild-type, mutant, or complemented strain and sterile PBS (200 μL). **(B)** RAW 264.7 cells were incubated with wild-type, mutant, or complemented strain at an M.O.I. of 10 for 3 to 12 h. IL-6 levels in cell culture supernatants were measured using ELISA. Data are expressed as mean ± SD from three independent experiments. *P < 0.05, **P < 0.01, ***P < 0.001.

All mice infected with the wild-type strain *C48-1* (50 CFUs/mice) died within 24 h, whereas the *C48-ΔPMCNA_RS00975* mutant (5 × 10^3^ CFUs/mice to 5 × 10^5^ CFUs/mice) could not cause any death during the trial on the seventh day of post-infection. The survival ratios (100%) of mice infected with the *C48-ΔPMCNA_RS00975* mutant were significantly increased (5 × 10^5^ CFUs/mice), and those of mice infected with the complemented strain *C48-ΔPMCNA_RS00975C* were partially regained to 60% (5 × 10^4^ CFUs/mice) and 50% (5 × 10^5^ CFUs/mice) (**Figure 2A**). In addition, all mice in the control group were injected with PBS and survived during the 7 consecutive observation days. Furthermore, a significant reduction was observed in IL-6 secretion by RAW264.7 cells (**Figure 2B**) exposed to the *C48-ΔPMCNA_RS00975* mutant strain compared with those exposed to wild-type and complemented strains 3 to 12 h post-infection. These results suggested that lack of *PMCNA_RS00975* markedly attenuated the virulence of *P. multocida C48-1* strain.

### 3.4 Purification and immunogenicity of rPMCNA_RS00975 protein

DNA sequencing confirmed the successful construction of the pPRO-EXHTb-*PMCNA_RS00975* recombinant expression vector. The rPMCNA_RS00975 protein expression induced with 0.5 mmol/L IPTG was largely present in the supernatant at 16°C (**Supplementary Figure S2A**). Further, the purified rPMCNA_RS00975 protein was confirmed by western blotting using an anti-His monoclonal antibody (**Supplementary Figure S2B**) or anti-*P. multocida* rabbit serum (**Supplementary Figure S2C**). These results indicated that rPMCNA_RS00975 protein is immunogenetic and expressed during *P. multocida* infection.

### 3.5 Subcellular localization of the PMCNA_RS00975 protein

**Figure 3:**
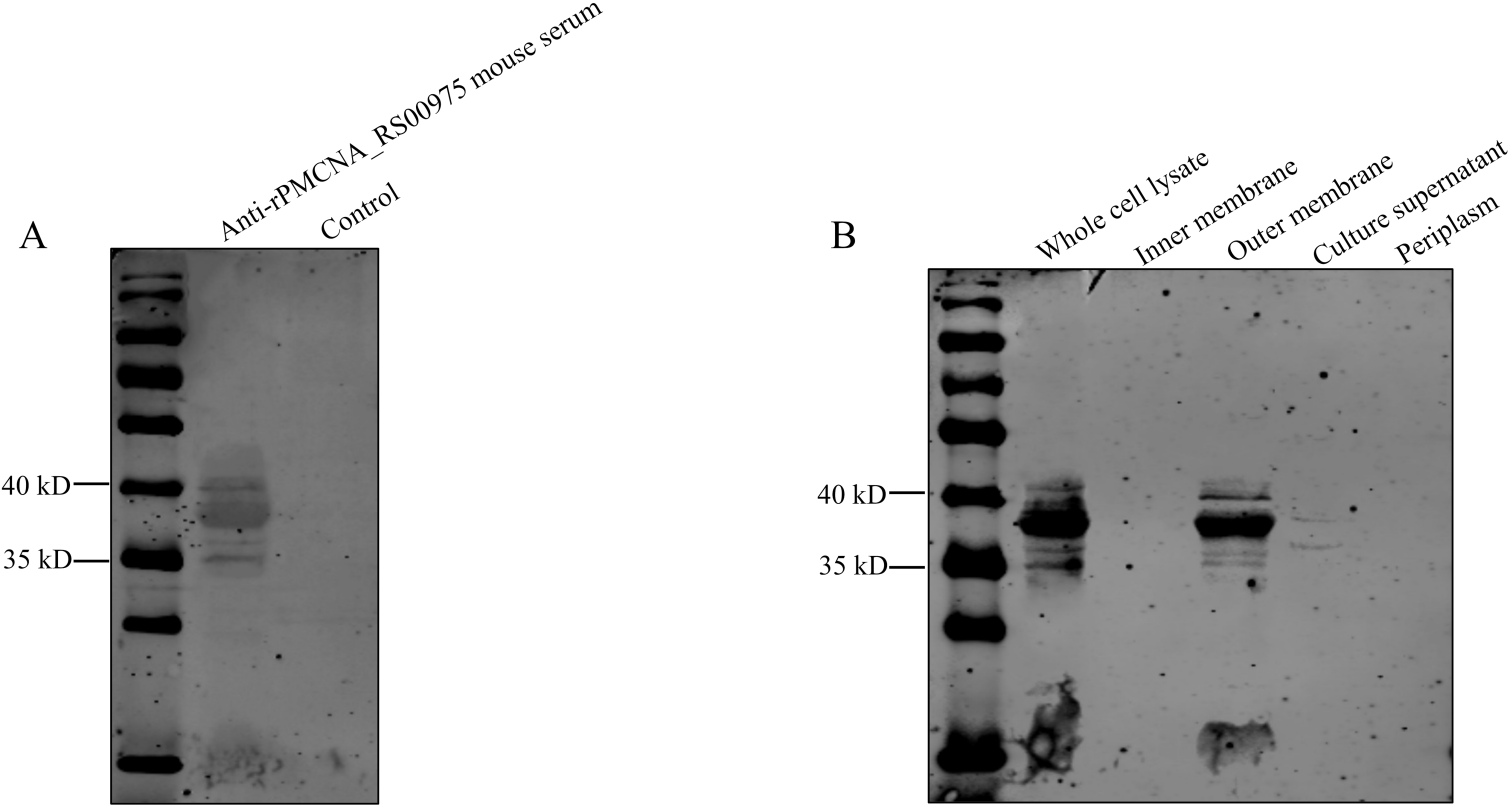
Subcellular localization of PMCNA_RS00975 protein. **(A)** The rPMCNA_RS00975 proteins reacts with the anti-rPMCNA_RS00975 mouse serum. **(B)** Subcellular localization of the rPMCNA_RS00975 protein was analyzed by western blotting using the anti-rPMCNA_RS00975 sera at a dilution of 1:1,000. The experiments were performed at least thrice.

To determine the localization of the PMCNA_RS00975 protein in *P. multocida C48-1*, cell fractions were isolated and analyzed by western blotting using the anti-rPMCNA_RS00975 mouse serum. The rPMCNA_RS00975 protein could be identified by the anti-rPMCNA_RS00975 mouse serum (**Figure 3A**). Additionally, the PMCNA_RS00975 protein was detected largely in the whole-cell lysate, cell outer membrane fraction, and the weak bands in the cell culture supernatant (**Figure 3B**), indicating that the PMCNA_RS00975 protein is located at the outer membrane of *P. multocida*.

### 3.6 rPMCNA_RS00975 induces IL-6 secretion of macrophages via the ERK1/2 and NF-κB signaling pathways

**Figure 4:**
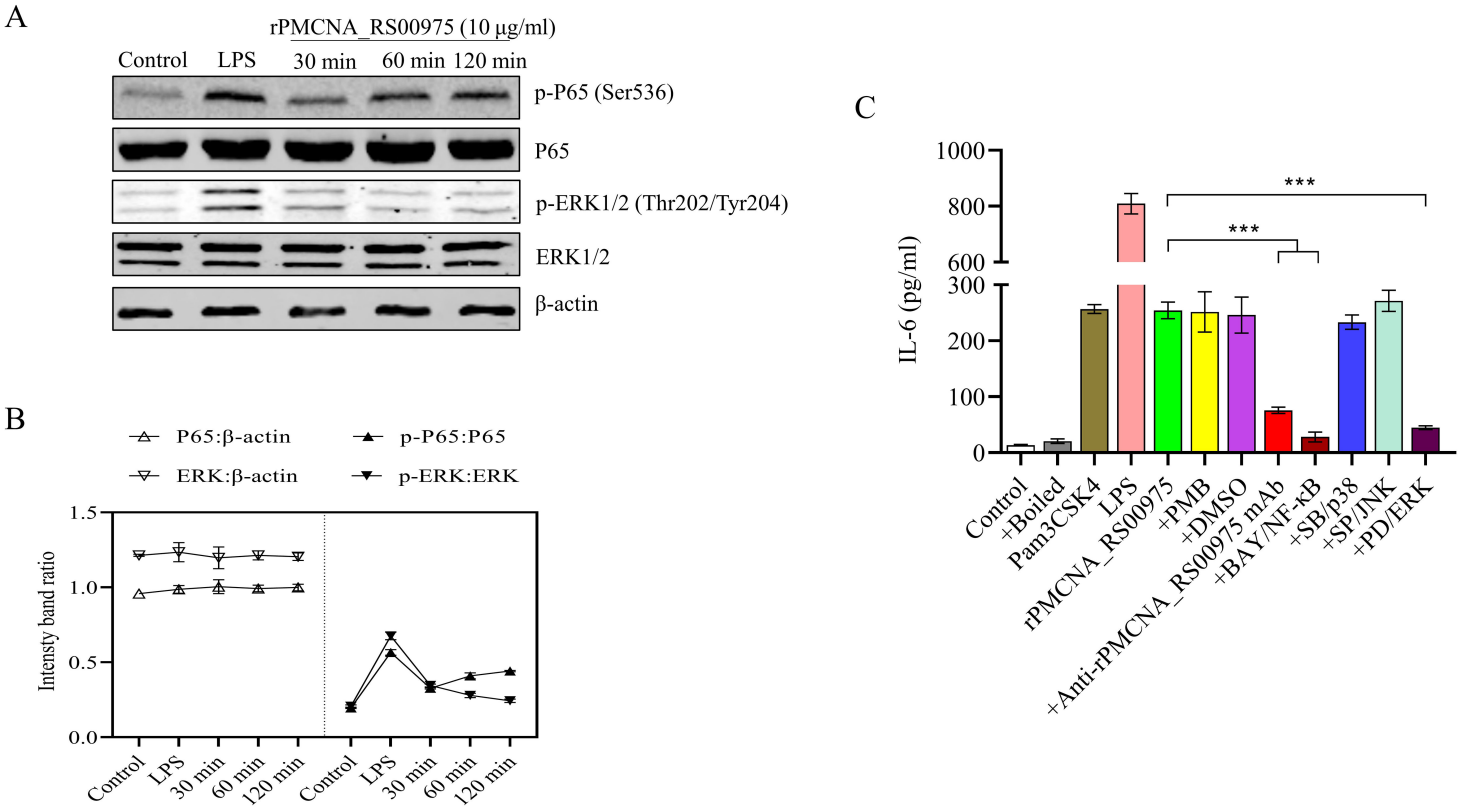
The rPMCNA_RS00975 protein induced the phosphorylation of ERK1/2 and P65. **(A)** RAW264.7 cells were incubated with rPMCNA_RS00975 (10 μg/mL), and the phosphorylation/activation status of ERK1/2 and p65 was analyzed by western blotting for the indicated times (30 min to 120 min). **(B)** Intensity bands ratio of activation status of p65 and ERK1/2 to β-actin was quantified using the ImageJ software. **(C)** rPMCNA_RS00975 was pretreated with polymyxin B (+PMB) and boiled (+Boiled) at 100℃ for 10 min to confirm that the activation of macrophages was because of rPMCNA_RS00975 and not LPS. rPMCNA_RS00975 was pretreated with mouse anti-rPMCNA_RS00975 serum (+anti-rPMCNA_RS00975) at 37°C for 1 h to confirm its neutralizing activity. RAW264.7 cells were pretreated for 30 min with PD98059 (+PD; 20 μM), SB203580 (+SB; 20 μM), SP600125 (+SP; 20 μM), BAY 11-7082 (+BAY; 20 μM), or dimethyl sulfoxide (0.01%) (+DMSO), and stimulated with the rPMCNA_RS00975 protein (10 μg/mL) for 6 h. The levels of IL-6 in the supernatants of RAW264.7 cells were measured by ELISA. Data are representative of at least three independent experiments. ***P< 0.001.

To investigate the function of the recombinant rPMCNA_RS00975 protein in inducing pro-inflammatory cytokines, the activation of the MAPK or NF-κB signaling pathways was analyzed by western blotting. Furthermore, the phosphorylation status of ERK1/2 and p65 was detected after the rPMCNA_RS00975 protein (10 μg/mL) was exposed to RAW264.7 cells. The results demonstrated the rPMCNA_RS00975 protein induced the phosphorylation status of ERK1/2 and p65 in RAW264.7 cells after 30 to 120 min (**Figure 4A**). The amount of p-p65 decreased over time, whereas the amount of p-p65 reached its peak at 30 min; the amount of p-ERK1/2 increased over time, whereas the amount of p-ERK1/2 reached their peak at 120 min (**Figure 4B**). These results indicated that the rPMCNA_RS00975 protein activated the NF-κB and ERK1/2 signaling pathways in macrophages. As expected, the IL-6 by LPS and Pam3CSK4 activities were significantly decreased in RAW264.7 cells. Furthermore, the expression of IL-6 was significantly inhibited by the inhibitors of p65 (BAY 11-7082) and ERK (U0126) but not by those of p38 (SB202190) and JNK (SP600125) (**Figure 4C**). These results proved that rPMCNA_RS00975 induced the secretion of IL-6 via the NF-κB and MAPK signaling pathways.

### 3.7 rPMCNA_RS00975-induced IL-6 expression by macrophages in a TLR2-dependent mechanism

**Figure 5:**
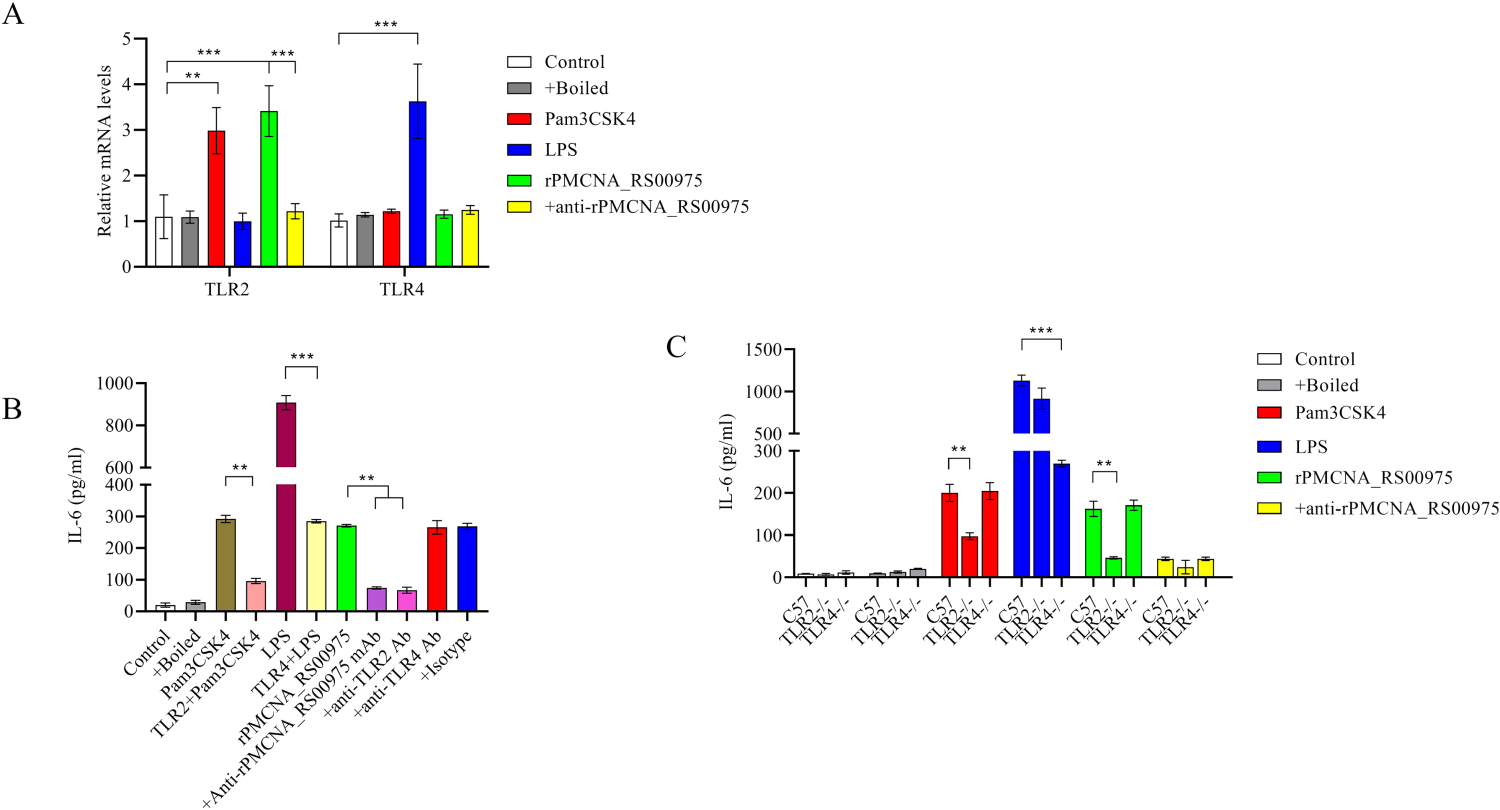
Secretion of IL-6 in response to rPMCNA_RS00975 protein is dependent on TLR2. rPMCNA_RS00975 was pretreated with mouse anti-rPMCNA_RS00975 serum (+anti-rPMCNA_RS00975) at 37°C for 1 h to confirm its neutralizing activity, or pretreated with boiled (+Boiled) at 100℃ for 10 min to confirm that the activation of macrophages was because of rPMCNA_RS00975 and not LPS. **(A)** TLR4 mRNA levels were determined by qRT-PCR after RAW264.7 macrophages stimulated with the rPMCNA_RS00975 protein (10 μg/mL) for 3 (h) **(B)** RAW264.7 cells were pretreated for 1 h with anti-TLR2 (5 μg/mL) (+ anti-TLR2), anti-TLR4 (5 μg/mL) (+anti-TLR4) or isotype (+Isotype) before stimulation with the rPMCNA_RS00975 protein (10 μg/mL) for 6 h. The levels of IL-6 in the culture supernatants of RAW264.7 cells were determined by ELISA. **(C)** Peritoneal macrophages from TLR2^−/−^, TLR4,^−/−^ and wild-type mice were incubated with Pam3CSK4 (100 ng/mL), LPS (100 ng/mL), or the rPMCNA_RS00975 protein (10 μg/mL) for 6 h. The levels of IL-6 in the supernatants of cells were measured by ELISA. Data are expressed as means ± SD from three separate experiments. **P< 0.01, ***P< 0.001.

rPMCNA_RS00975 protein induced IL-6 to promote the identification of the signaling for this protein and trigger the secretion of pro-inflammatory cytokines. The mRNAs of *TLR2* but not those of *TLR4* were upregulated after stimulating the peritoneal macrophages with rPMCNA_RS00975 (**Figure 5A**). Further, RAW264.7 cells were pretreated with or without blocking antibodies against TLR2, TLR4, or an isotype control antibody for 1 h before stimulation with rPMCNA_RS00975, LPS, or Pam3CSK4. LPS and Pam3CSK4 were used as positive controls to demonstrate the blocking effects of anti-TLR4 and anti-TLR2 antibodies. The rPMCNA_RS00975 protein-induced production of IL-6 in RAW264.7 cells was inhibited by specific antibodies against TLR2, but not by specific antibodies against TLR4 (**Figure 5B**). In addition, compared with the release of IL-6 in peritoneal macrophages isolated from C57BL/6N mice, the release of IL-6 was inhibited in those isolated from TlR2^−/−^ mice, but not in those isolated from TLR4^−/−^ mice (**Figure 5C**). These results indicated that rPMCNA_RS00975 protein-induced IL-6 proinflammatory cytokines were mediated via the TLR2 signaling.

## 4 Discussion

Virulence factors (e.g., toxins) have been implicated in bacterial pathogenesis and function via horizontal gene transfer (Brüssow et al., 2004; Doakes and Koskella, 2024; Ledvina and Whiteley, 2024). PMT is the only toxin protein of *P. multocida*, produced by the toxin-producing *P. multocida* (mostly capsular types D and A) (Shirzad Aski and Tabatabaei, 2016; Prajapati et al., 2024). These bacteria express the *toxA* gene that is located in the lytic phage sequence (Siddaramappa, 2021). The presence of *toxA* on a lysogenic phage may allow both the expression and release of the toxin during disease progression (Bethe et al., 2009). In addition, pathogenic bacteria require iron-associated genes to retrieve iron from animal hosts during the infection and disease process (Choby and Skaar, 2016; Donegan, 2022). For example, the B739_1343 of *Riemerella anatipestifer* is a putative TonB-dependent receptor involved in the utilization of inorganic ions. A study reported that the median lethal dose (LD_50_) of deletion of B739_1343 mutant strain increased more than 10^4^-fold compared with that of the wild-type strain RA-CH-1 (Liu et al., 2018). *P. multocida* has evolved to express several iron-uptake genes to acquire pathways in response to iron sources, such as hemoglobin, transferrin, ferritin, or ferric citrate, particularly the outer membrane iron receptors and transporters (Jatuponwiphat et al., 2019; Uchida et al., 2022). Similarly, the *PMCNA_RS00975* gene is located in the lysogenic phage sequence of the *C48-1* strain and encodes the encapsulating protein that is possibly implicated in iron utilization. However, further research is warranted, including the detailed secretion mechanism of PMCNA_RS00975 protein, and whether *PMCNA_RS00975* affects the iron utilization of wild-type *C48-1*.

Encapsulin nanocompartments are a family of proteinaceous metabolic compartments that are widely distributed in bacteria and archaea (Giessen, 2022; McDowell and Hoiczyk, 2022; Quinton et al., 2023). The family shares a common architecture, including an icosahedral shell composed of a oligomeric assembly of a protein associated with the HK97 bacteriophage capsid protein gp5 (Hawkins et al., 2023). Gp5 is reported to be a capsid protein expressed from the HK97 bacteriophage and composed of a 66 nm diameter icosahedral shell consisting of 420 subunits (He et al., 2016; Wang et al., 2022; Hawkins et al., 2023). However, encapsulin shell proteins are composed of 32 nm icosahedra with 180 subunits in *Pyrococcus furiosus* and *Myxococcus xanthus* (Altenburg et al., 2021). The *T. maritim*a encapsulin has a smaller structure and is composed of a 25 nm, 60-subunit icosahedron, and the high homologous encapsulin shell proteins with gp5 have evolved from a common evolutionary ancestor for the above-mentioned proteins (Altenburg et al., 2021). Research has demonstrated the genes encoding dye-dependent peroxidase family enzymes are mostly located upstream of the genes encoding encapsulin proteins, also known as EncFtn (Dhankhar et al., 2020; Jones et al., 2024). A previous study has reported that EncFtn proteins can be isolated by encapsulin protein in *M. xanthus*, and form an ‘iron-megastore’ to protect bacteria from oxidative stress (McHugh et al., 2014; Altenburg et al., 2021). The PMCNA_RS00975 is speculated to be encapsulating protein, affecting iron metabolism; antioxidant stress requires to be further explored.

Microorganism are recognized by pattern recognition receptors (PRRs) of the antigen-presenting cell, and certain proteins from microorganism bind to different receptor adapter proteins in the cytoplasm to activate the MAPK and NF-κB and other signal transduction pathways (Barnett et al., 2023). NF-κB and MAPK are important components that regulate the expression of pro-inflammatory cytokines mediators. NF-κB exists in the cytoplasm as a complex with IκB, which, when activated, phosphorylates and degrades IκB, and NF-κB enters the nucleus and affects the transcription of related genes (Chen et al., 2023; Guo et al., 2024). The MAPK signal transduction pathway includes ERK1/2, stress-activated protein kinases JNK and p38, which can be activated alone or simultaneously (Nandi and Aroeti, 2023). The *P. multocida* porin outer membrane protein can induce the production of pro-inflammatory cytokines IL-1α, IL-6, TNF-α, INF-γ, and IL-12, but not IL-4 and IL-10 in mouse splenocytes (Hatfaludi et al., 2010; He et al., 2020). Porins of other gram-negative bacteria can activate the NF-κB signaling pathways to produce cytokines through TLR2 receptors; however, the OmpH activation mechanism of *P. multocida* remains elusive (Hatfaludi et al., 2010). This study indicates that the p65 and ERK1/2 MAPK signaling pathways contribute to the release of IL-6 induced by rPMCNA_RS00975 protein in macrophages. However, it is unclear whether other signaling pathways, such as mTOR, are involved in this process.

The constructed *PMCNA_RS00975* mutant was significantly attenuated to mice compared with the wild-type strain, indicating that this protein is an important virulence factor for *P. multocida*. In addition, the *PMCNA_RS00975* gene deletion mutant of *P. multocida C48-1* strain was constructed by positive selection techniques, leading to a *kan*-resistance expression cassette remaining in the genome. *Ng*Ago technology, without molecular marker-free in the genome, has been recently used to successfully construct multiple mutants of *P. multocida* (Fu et al., 2019). We believe that the findings of this study will provide a good basis for the development of an attenuated vaccine against *P. multocida*.

## 5 Conclusion

In summary, our results showed that the PMCNA_RS00975 protein, which is encoded by one of the gene clusters from prophage island of pathogenic strain *C48-1*, is located at outer membrane and contributes the virulence of *P. multocida*. Furthermore, the rPMCNA_RS00975 protein was found to be able to induce the release of the IL-6 pro-inflammatory cytokines triggered by the TLR2 in the macrophages via the NF-κB and ERK1/2 signaling pathways. These results thus provide a new perspective for pathogenicity and the pro-inflammatory cytokines of *P. multocida*.

## Data Availability

The raw data supporting the conclusions of this article will be made available by the authors, without undue reservation.

## Supplementary material

Supplementary Figure 1 Construction and confirmation of mutant and complemented strains. **(A)** Construction strategies of the *C48-ΔPMCNA_RS00975* mutant. **(B)** Confirmation of the *C48-ΔPMCNA_RS00975* mutant and complemented strain *C48-ΔPMCNA_RS00975C* by PCR. M: DNA marker; Lanes 1–4: Wild-type *C48-1* (positive control) and control; Lanes 5–8: *C48-ΔPMCNA_RS00975* mutant and control; Lanes 9–12: complemented strain *C48-ΔPMCNA_RS00975C* and control. Lanes 1, 5, and 9: PCR production using primers KMT1-T7/KMT1-SP6 (positive control); lanes 2, 6, and 10: PCR production using primers PPm00975-JD-F/PPm00975-JD-R; lanes 3, 7, and 11: PCR production using primers PPm00975-EF/*Spec*-R; 4, 8, and 12: negative control. **(C)** Growth curves of *C48-1*, *C48-ΔPMCNA_RS00975* mutant, and complemented strain *C48-ΔPMCNA_RS00975C*. Results shown are representative of three independent experiments.

Supplementary Figure 2 Purification and immunogenicity of rPMCNA_RS00975 protein. **(A)** SDS-PAGE analysis of rPMCNA_RS00975 protein. Lane M, protein markers. Lane 1, purified rPMCNA_RS00975 protein. Lane 2, supernatant of recombinant *E. coli* expressing rPMCNA_RS00975 protein. Lane 3, lysis solution of recombinant *E. coli* expressing rPMCNA_RS00975 protein. **(B)** The rPMCNA_RS00975 protein reacted with the anti-His antibody. **(C)** The rPMCNA_RS00975 protein reacted with the antiserum obtained from rabbits infected with *P. multocida*. Experiments were performed at least thrice.
