# Supplementary figures and images for: PMCNA_RS00975 activates NF-κB and ERK1/2 through TLR2 and contributes to the virulence of Pasteurella multocida
Source: Front Cell Infect Microbiol. 2024 Oct 15;14:1469304. doi: 10.3389/fcimb.2024.1469304 (PMC11518796; doi:10.3389/fcimb.2024.1469304)

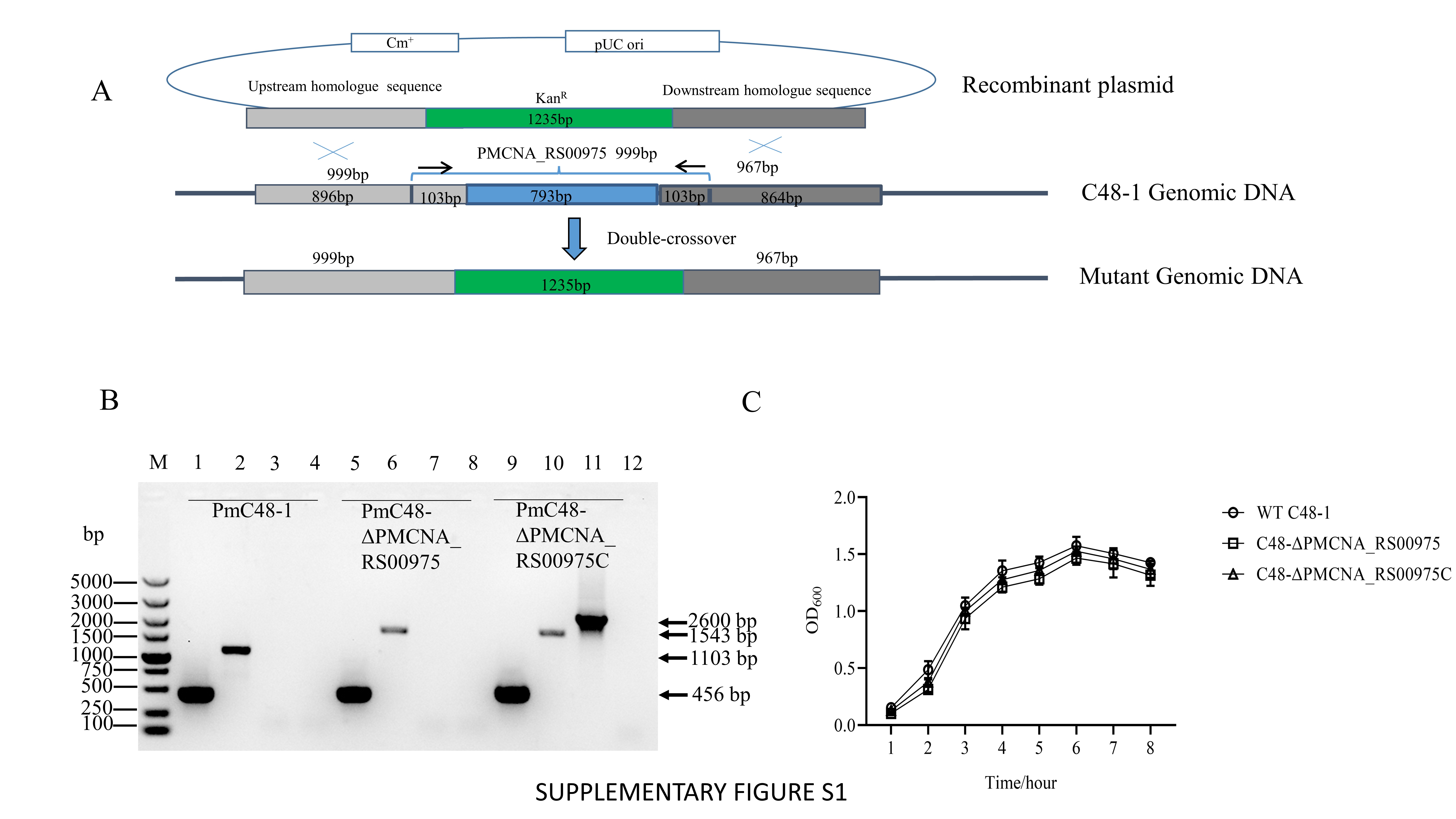

Supplement: Supplementary Figure 1 — Construction and confirmation of mutant and complemented strains. (A) Construction strategies of the C48-ΔPMCNA_RS00975 mutant. (B) Confirmation of the C48-ΔPMCNA_RS00975 mutant and complemented strain C48-ΔPMCNA_RS00975C by PCR. M: DNA marker; Lanes 1–4: Wild-type C48-1 (positive control) and control; Lanes 5–8: C48-ΔPMCNA_RS00975 mutant and control; Lanes 9–12: complemented strain C48-ΔPMCNA_RS00975C and control. Lanes 1, 5, and 9: PCR production using primers KMT1-T7/KMT1-SP6 (positive control); lanes 2, 6, and 10: PCR production using primers PPm00975-JD-F/PPm00975-JD-R; lanes 3, 7, and 11: PCR production using primers PPm00975-EF/Spec-R; 4, 8, and 12: negative control. (C) Growth curves of C48-1, C48-ΔPMCNA_RS00975 mutant, and complemented strain C48-ΔPMCNA_RS00975C. Results shown are representative of three independent experiments. [file Image1.jpg]

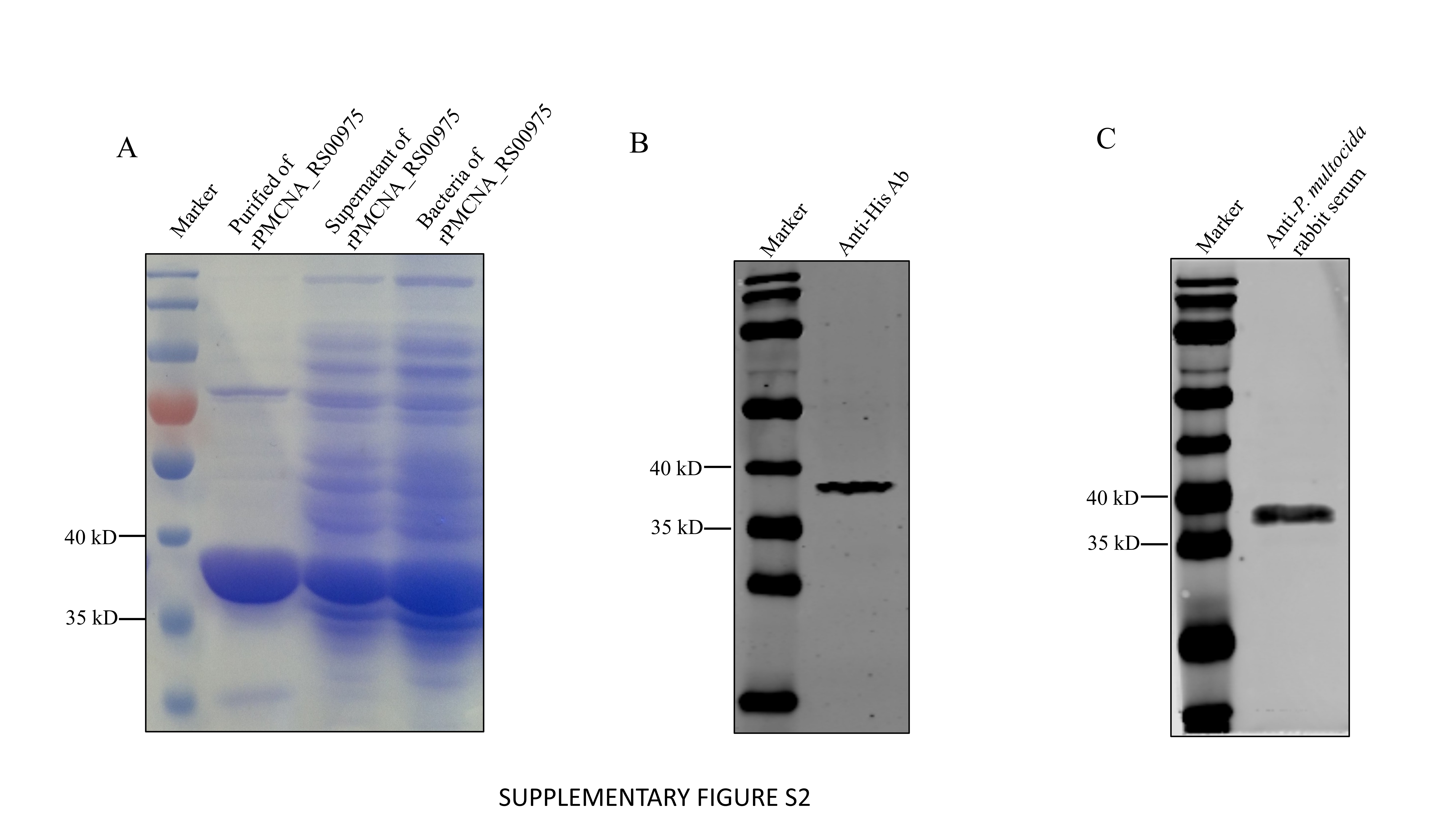

Supplement: Supplementary Figure 2 — Purification and immunogenicity of rPMCNA_RS00975 protein. (A) SDS-PAGE analysis of rPMCNA_RS00975 protein. Lane M, protein markers. Lane 1, purified rPMCNA_RS00975 protein. Lane 2, supernatant of recombinant E. coli expressing rPMCNA_RS00975 protein. Lane 3, lysis solution of recombinant E. coli expressing rPMCNA_RS00975 protein. (B) The rPMCNA_RS00975 protein reacted with the anti-His antibody. (C) The rPMCNA_RS00975 protein reacted with the antiserum obtained from rabbits infected with P. multocida. Experiments were performed at least thrice. [file Image2.jpg]
