# Supplementary material for: PMCNA_RS00975 activates NF-κB and ERK1/2 through TLR2 and contributes to the virulence of Pasteurella multocida
Source: Front Cell Infect Microbiol. 2024 Oct 15;14:1469304. doi: 10.3389/fcimb.2024.1469304 (PMC11518796; doi:10.3389/fcimb.2024.1469304)
Supplement: Supplementary file 3 [file Table1.docx]

**Table S1.** Different bacteria used to construct evolutionary trees in this study

| Production | Accession number | Pathogen | Strain |
| --- | --- | --- | --- |
| Encapsulin | WP_142499566.1 | *klebsiella pneumoniae* | ATCC 43816 |
| Encapsulin | WP_189116341.1 | *Klebsiella michiganensis* | E718 |
| Encapsulin | MBI6135081.1 | *Serratia marcescens* | SER00255 |
| Encapsulin | WP_038910933.1 | *Dickeya dadantii* | 3937 |
| Encapsulin | WP_200011321.1 | *Citrobacter freundii* | ATCC 8090 |
| Encapsulin | WP_129995503.1 | *Enterobacter cloacae* | ATCC 13047 |
| Encapsulin | WP_199557630.1 | *Enterobacter roggenkampii* | CHS 79 |
| Encapsulin | WP_175273061.1 | *Kosakonia sacchari* | SP1 |
| Encapsulin | WP_187497010.1 | *Pantoea sp.* | Psp39-30 |
| Encapsulin | WP_167864680.1 | *Erwinia rhapontici* | ATCC 29283 |
| DUF6260 family protein | WP_064558964.1 | *Buttiauxella brennerae* | ATCC 51605 |
| Encapsulin | EHK8991086.1 | *Escherichia coli* | FSIS12139128 |
| Encapsulin | MBO6227484.1 | *Shewanella sp.* | RGIG9240 |
| PM0194 | WP_064702788.1 | *Pasteurella multocida* | C48-1 |
| DUF6260 family protein | WP_064702788.1 | *Pasteurella multocida* | Pm70 |
| Encapsulin | WP_006251229.1 | *Mannheimia haemolytica* | D153 |
| Encapsulating for peroxidase | MWQ04288.1 | *Glaesserella parasuis* | H222 |
| Encapsulin | WP_176808249.1 | *Mannheimia pernigra* | 16CN0041 |
